# Salivirus in Children and Its Association with Childhood Acute Gastroenteritis: A Paired Case-Control Study

**DOI:** 10.1371/journal.pone.0130977

**Published:** 2015-07-20

**Authors:** Jie-mei Yu, Yuan-yun Ao, Na Liu, Li-li Li, Zhao-jun Duan

**Affiliations:** Institute for Viral Diseases Control and Prevention, China CDC, Beijing, China; University of California, San Francisco, UNITED STATES

## Abstract

Salivirus was recently discovered in children with gastroenteritis and in sewage. Though a causative role for salivirus in childhood gastroenteritis was suggested in the previous study, the relationship between salivirus and acute gastroenteritis has not yet been clearly clarified. The sewage strain reported by Ng, although represented by incomplete genome sequencing data, was distinct from previously reported saliviruses, and had not previously been detected in humans. A case-control study examining 461 paired stool samples from children with diarrhea and healthy controls (1:1) was conducted in this study. Also, common diarrheal viruses were detected and complete genome of a salivirus was determined. Results showed that salivirus was detected in 16 (3.5%) and 13 (2.8%) of the case and control samples, respectively; no differences in detection rates (*p*=0.571) or mean values of viral loads (*p*=0.400) were observed between the groups. Multivariate Cox regression revealed no association between salivirus and gastroenteritis (*p*=0.774). The data also demonstrated that salivirus infection did not exacerbate clinical symptoms of gastroenteritis in children. Furthermore, complete genome sequence of a salivirus recovered from the feces of a child with diarrhea (i.e., SaliV-FHB) shared a 99% nucleotide identity with the sewage strain. In conclusion, a paired case-control study did not support a causative role for salivirus strains detected in this study with pediatric gastroenteritis. This study also demonstrated that all known saliviruses can be detected in the feces of children with or without gastroenteritis.

## Introduction

Acute gastroenteritis remains one of the leading causes of death in children worldwide. Rotavirus (RV) and norovirus (NoV) are the most common viral pathogens, followed by astroviruses (AstV) and adenoviruses (AdV) [1–3]. Although the majority of gastroenteritis cases are associated with infection by known pathogens, up to 40% of cases are of unknown etiology [4, 5]. The role of unrecognized infectious agents, including viruses, as causes of gastroenteritis should be given more attention.

Salivirus, a member of the family *Picornaviridae*, was first reported in the feces of children with gastroenteritis in both the United States and Australia named as klassevirus in 2009 [6, 7]. Almost simultaneously, salivirus was discovered in the feces of children with non-polio acute flaccid paralysis in Nigeria [8]. Subsequently, both salivirus and klassevirus were classified as belonging to the novel genus *Salivirus* in *Picornaviridae*. In 2013, the *Picornaviridae* Study Group further defined salivirus and klassevirus as being from the same species (http://www.ictvonline.org/virusTaxonomy.asp), due to the high similarity of their genomes.

Saliviruses in previous studies were found in stool or sewage [6–9], and it was considered to be associated with acute gastroenteritis by molecular epidemiology, and seroepidemiology study also showed proof of infection [6, 10–11]. However, due to the low detection rate of the virus, and to the small sample sizes employed in these previous studies (which also did not include a strict, case-control study), the relationship between salivirus and acute gastroenteritis has not yet been clearly established. The different varieties of salivirus are highly similar, with the exception of SaliV-SewBKK (detected in sewage), the complete genome of which has not yet been acquired [9]. Here a strict 1:1 paired case-control study was conducted to investigate the prevalence of salivirus in children and the relationship between salivirus and acute gastroenteritis. During the process, a salivirus (SaliV-FHB), most similar (99% shared nucleotide [nt] identity) to SaliV-SewBKK, was detected in fecal samples and its complete genome sequence was acquired.

## Materials and Methods

### Study area, participants and design

This study was conducted in two developing areas of China, one in the north (Lulong, Hebei province), and the other in the south (Liuyang, Hunan province). A case-control study was employed: inpatient and outpatient children with diarrhea and healthy children under 5 years of age were recruited and paired according to age (age groups: 0–5, 6–11, 12–23, 24–35, 36–47 and 48–59 months) and sex, each age group comprised equal numbers of case and control subjects. The maximum time interval between matched case and control sampling was 14 days. Between May 2011 and January 2013, 239 and 222 pairs of fecal samples were collected in Lulong and Liuyang, respectively.

### Definitions and inclusion criteria for case and control subjects

Case subjects were diagnosed with diarrhea by a pediatrician. Diarrhea was defined as at least three loose stools in the previous 24–72 h. Patients were excluded from the study if they had another diagnosed illness, such as pneumonia. Control subjects were healthy children who did not have diarrhea, fever, vomiting or a respiratory illness during the previous week. Control subjects received follow-up by telephone; children with the above-listed clinical symptoms during the week following the initial examination were also excluded.

### Sample processing

For the inpatients the stool specimens were collected within 24 h of hospitalization, and for the outpatient the stool specimens were collected when they visited the hospital. All stool specimens were stored at -70°C.

### Ethics Statement

In this study, physicians signed on the consent form on behalf of the guardians after getting their verbal approval for each child who provided a specimen. The consent procedure was approved by the Ethics Committee of the National Institute for Viral Disease Control and Prevention, China CDC, according to Chinese ethics laws and regulations and the study protocol was approved by the Ethics Committee of the National Institute for Viral Disease Control and Prevention, China CDC, according to Chinese ethics laws and regulations.

### Nucleotide extraction and reverse transcription

Total nucleic acids were extracted from 10% fecal suspensions. First-strand cDNAs were synthesized using the reverse transcriptase Superscript II (Life Technologies, USA). The reaction was initiated by incubation at 42°C for 1 h, then at 99°C for 5 min; it was subsequently maintained at 4°C.

### Detection of common diarrhea-related viruses

All specimens were tested for RV, NoV and sapovirus (SaV) (NoV and SaV belong to calicivirus, the positive rates of them are calculated as a whole in the manuscript), AstV and AdV by real-time PCR using the primers, probes, and reaction conditions reported in previous studies [12–15].

### Salivirus detection

Salivirus was detected using nested PCR amplification of a 414-base pair fragment located in the 5′ untranslated region (UTR) [10]. Positive and negative controls were included. Salivirus viral loads in positive samples were quantified by real-time PCR as developed and described in a previous study (Forward primer: 5′-TCTGCTTGGTGCCAACCTC-3′; Reverse primer: 5′-CCARGCACACACATGAGRGGATAC-3′ and probe: 5′-FAM- TGCGGGAGTGCTCT- MGB- NFQ-3′) [16]. RNA run-off transcripts were used as quantitative reference.

### Complete genomic amplification

Primers were initially designed based on the sequence acquired by nested PCR. Further synthesis was based on newly amplified sequences. The extreme 5′ and 3′ ends of the genome were determined using a SMART RACE cDNA amplification Kit (Clontech, USA). Long fragments were further amplified using ExTaq DNA polymerase (Takara, Japan) for final sequence confirmation. Sequences were assembled and edited manually to produce the final sequence of the viral genome.

### Sequencing and phylogenetic analysis

Phylogenetic analyses were performed using nucleotide sequences according to the neighbor-joining method, and were subsequently subjected to bootstrap analysis with 1000 replicates to determine the reliability values at each internal node. Trees constructed using the MEGA software (ver. 5.0).

### Statistical analysis

T-tests and χ^2^ tests were performed to assess the significance of viral loads and positivity rates of various viral pathogens in cases and controls. Associations between viral infection and gastroenteritis were assessed using a Cox regression analysis. Associations between clinical symptoms and viral infections were assessed using logistic regression. All statistical analyses were performed with the SPSS for Windows software package (ver. 16.0; SPSS, Chicago, IL).

## Results

### Common viral agents in children

**Fig 1:**
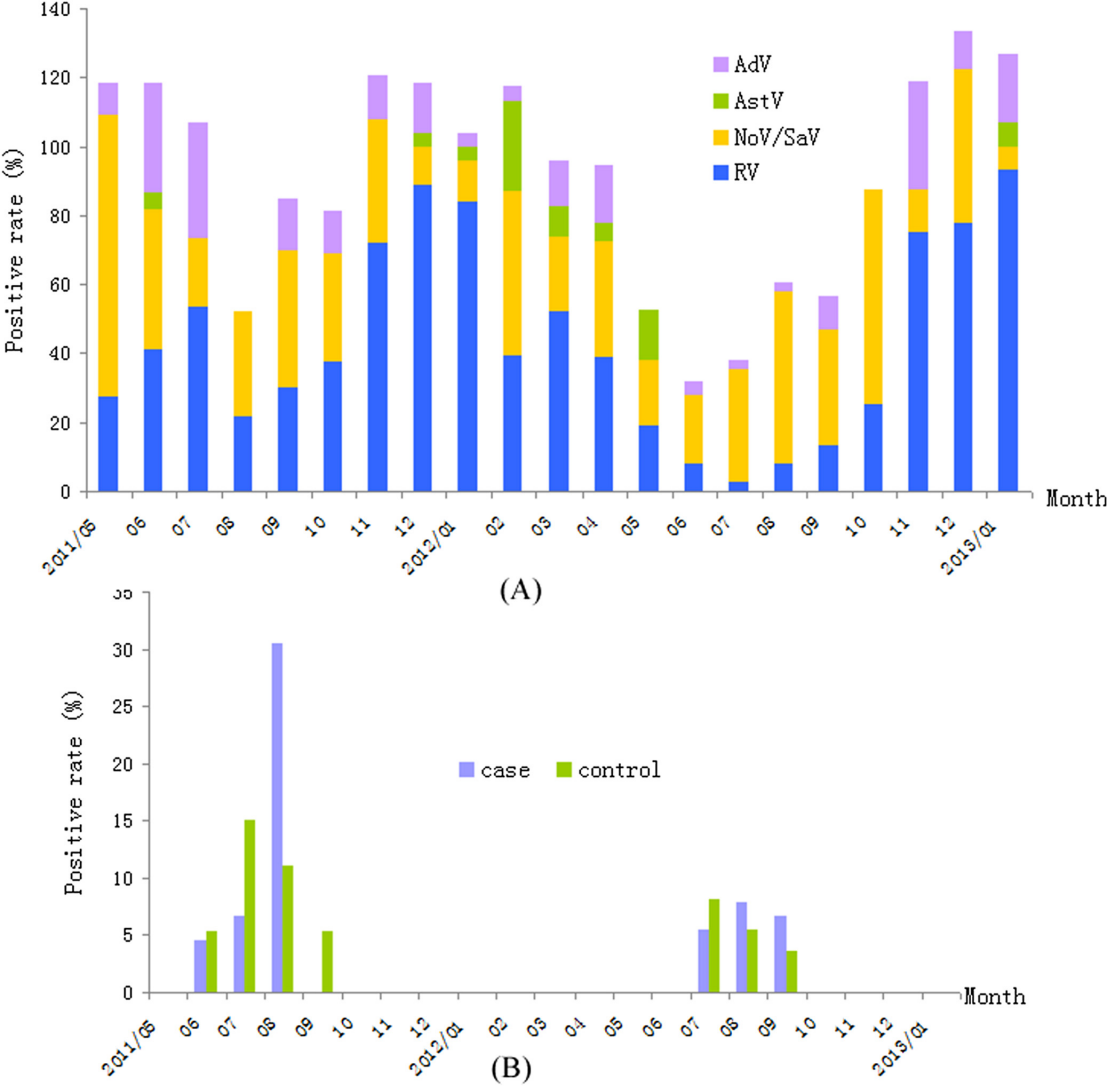
A. The monthly distribution of major viral pathogens in pediatric patients with gastroenteritis. RV peaked during the winters in the two years, NoV/SaV, AstV and AdV exhibited no marked seasonal distribution. B. The monthly distribution of salivirus in pediatric patients with gastroenteritis. The viruses in the case and control groups were detected in the hottest days of the year. Note: Y axis representing accumulating positive rate greater than 100% is due to some of the specimens were positive for more than one virus.

The ages of children comprising the 461 subject pairs ranged between 1 and 58 months (mean age = 11.67 ± 8.42 months). The majority of the children (93.93%) were 1–24 months of age. The ratio of females to males was 1.88:1. In the case group, RV was the most commonly detected virus (40.6%), followed by NoV/SaV (31.5%), AdV (10.9%) and AstV (3.3%); in the control group, NoV/SaV were the most-commonly detected viruses (14.3%), followed by AdV (2.6%), RV (1.7%) and AstV (1.3%). The monthly distribution of the viral pathogens are described in Fig 1A, which suggests that RV detection peaked during the two winters, whereas NoV/SaV, AstV and AdV exhibited no marked seasonal distribution.

### Detection of saliviruses

Nested PCR detected saliviruses in 16 (3.5%) case subjects and 13 (2.8%) control subjects. All viruses in the case and control groups were detected between June and September, a period containing the hottest days of the year. On colder days, the virus was not detected (Fig 1B). Of the 16 salivirus-positive case subjects, 7 were from the Hebei province (North China), and 9 were from the Hunan province (South China), 7 had fever and 3 were vomiting (Table 1). The χ^2^- test revealed no significant difference in detection rates by groups (*p* = 0.571) and by areas (*p* = 0.524). The age range of salivirus-positive case subjects was 1–9 months (median = 4 months; P25/P75 = 2/8 months), and 10–27 months (Median = 16 months; P25/P75 = 12/18 months) in the control group.

**Table 1:** Clinical characteristics, viral loads and related information of the 16 case children who were positive for salivirus.

Patient ID No.	Sex	Area	Duration of diarrhea, days	Age, Month	Coinfection	Fever	Frequency of diarrhea, per day	Vomiting	Viral load, copies
1113044	M	Hebei	N	3	RV,NoV/SaV,AdV	-	8	-	4.88E+02
1113059	F	Hebei	4	4	NoV/SaV	-	7	-	4.57E+04
1113065	M	Hebei	5	4	-	-	3	+	8.15E+01
1113066	M	Hebei	2	4	-	-	10	-	1.14E+00
1143016	M	Hunan	7	2	NoV/SaV	+	4	-	6.91E+05
1143032	F	Hunan	6	4	-	+	4	-	5.99E+02
1143033	F	Hunan	5	4	RV, NoV/SaV	+	3	-	5.04E+02
1143035	M	Hunan	9	4	NoV/SaV	+	10	-	1.70E+03
1143038	F	Hunan	5	4	RV	+	7	+	1.42E+03
1213129	M	Hebei	1	15	-	+	2	-	9.64E+03
1213139	M	Hebei	4	16	NoV/SaV	_	2	+	8.51E+02
1213147	M	Hebei	5	16	-	_	3	-	2.34E+04
1243022	F	Hunan	5	15	-	N	6	-	8.46E+00
1243057	M	Hunan	9	16	NoV/SaV	_	4	-	1.59E+04
1243073	M	Hunan	7	17	NoV/SaV	+	7	-	1.64E+00
1243074	F	Hunan	9	17	RV, NoV/SaV	N	3	-	2.22E+05

Note: Duration of diarrhea indicates the time from the admission date to the discharge date of hospitalization. M, male; F, female; N, no data; +, Present;-, absent.

**Fig 2:**
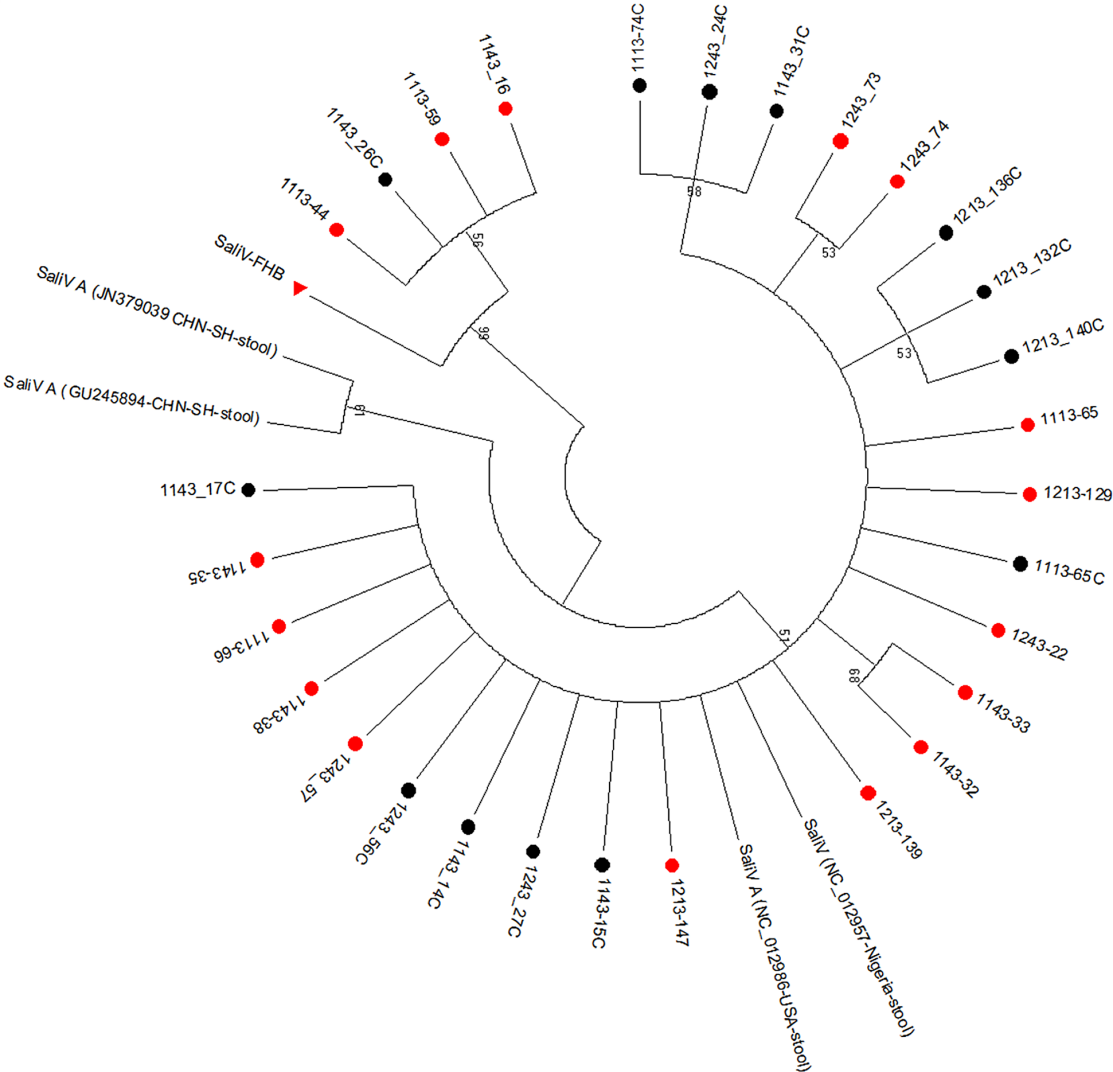
Phylogenetic relationships based on the 5′ UTR sequences amplified in this study and those of other saliviruses. The sequences acquired in this study were in the same lineage. Sequences acquired from cases are marked with“●” in red, while from healthy controls are marked with “●” in black. SaliV-HFB is marked with “▲” in red.

Of the 29-salivirus sequences, it was found that 4 (SaliV-FHB) were close to SaliV-SewBKK, with more than 95% shared nt identities; the other 25 sequences were close to salivirus A, with shared nt identities of between 98% and 99%. Of the four SaliV-FHB-positive samples, three were from the case group and one from the control group. Phylogenetic analysis of these sequences using the neighbor-joining method and 1,000 bootstrap replications showed they were from the same lineage (Fig 2). These sequences were deposited in GenBank under the accession number of KM051477-KM051505. In addition, among the 16 salivirus-positive children with diarrhea, 10 (62.5%) were coinfected with another diarrhea-related virus, among which NoV/SaV was the most common (*n = 9*) (Table 1).

### Complete genome sequencing of SaliV-FHB

Since previous reported SaliV-SewBKK was detected in sewage and its sequence is incomplete, we amplified the whole complete genome of SaliV-FHB in the study. A total of 10 fragments were amplified to generate new genome sequences and a final genome sequence (Primers used here were listed in S1 Table). The full-length genome sequence was deposited in GenBank under the accession number KM023140. The identified SaliV-FHB genome comprises 7968 bp (excluding the polyadenylated tract), with a 730-bp 5′ UTR, an open reading frame of 7098 nt (encoding a potential polyprotein precursor of 2166 amino acids), followed by a 140-bp 3′ UTR and poly (A) tail.

**Fig 3:**
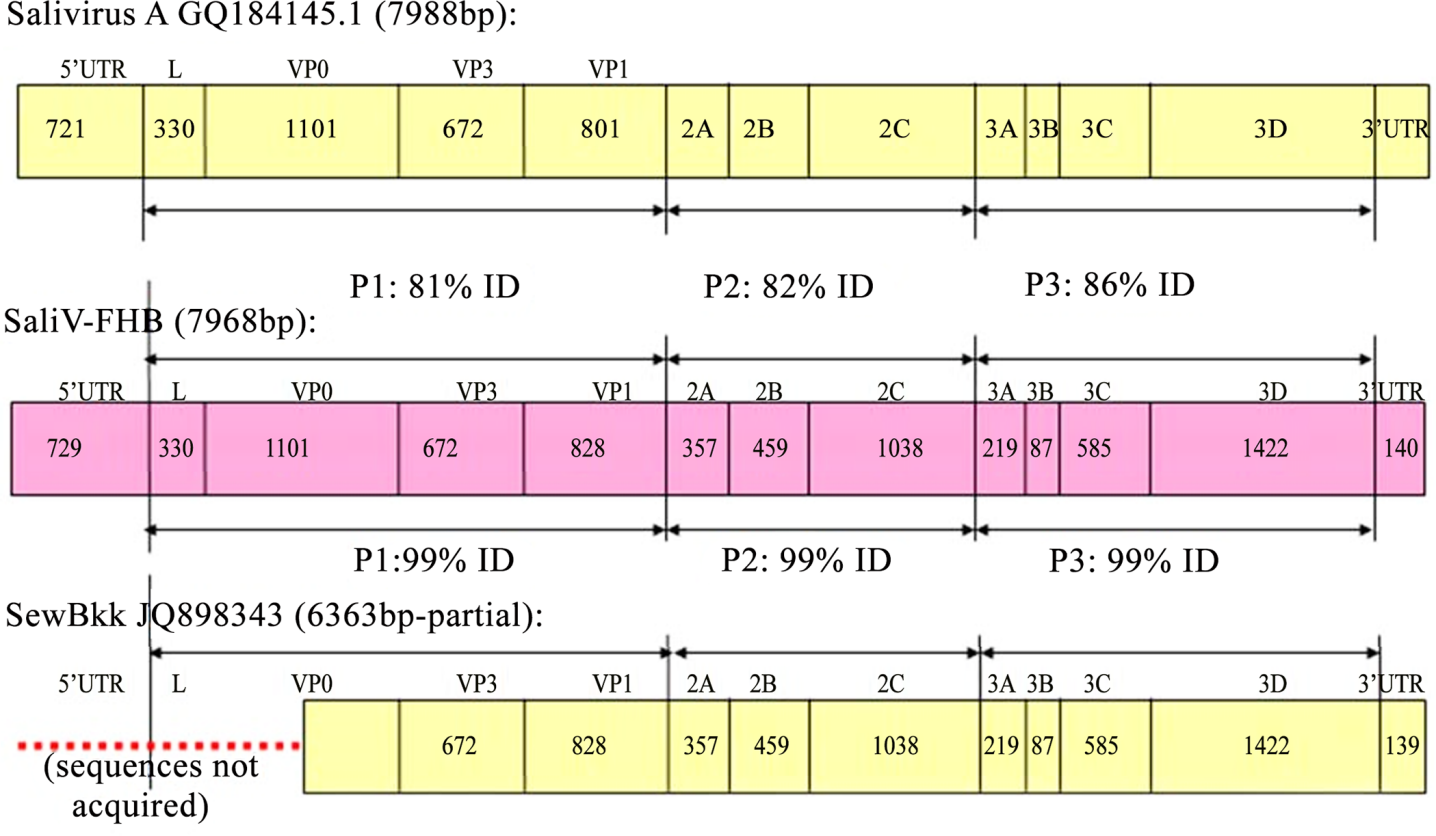
Compare of full genome sequences between SaliV-FHB and salivirus A and SaliV-SewBKK. P1 region of SaliV-FHB shared lowest nt identity to that of Salivirus A, while P1, P2 and P3 regions of SaliV-FHB shared 99% nt identities to those of SaliV-SewBKK. "ID" stands for identity.

The base usage of the SaliV-FHB genome was 17.54% A, 36.0% C, 21.44% G and 25.0% U, with a pyrimidine content of 61%, which is much higher compared with the other viruses of the *Picornaviridae* family (HPeV1: 47.1%; LV 145SL: 49.11%; SAFV: 51.0%; and SV19: 48.1%). BLASTn analysis revealed that the full genome of SaliV-FHB exhibited 99% shared nt identity with SaliV-SewBKK, with only 79% query coverage, and 83% shared nt identity with Salivirus A, with 99% query coverage. Further, P1 region of SaliV-FHB shared lowest nt identity to that of Salivirus A, while P1, P2 and P3 regions of SaliV-FHB all shared 99% nt identities to those of SaliV-SewBKK (Fig 3). Compared to other saliviruses, SaliV-FHB is characterized by a marked difference in the 5′ UTR: a redundant sequence of 21/22-bp length was observed (position 214/5-236bp; sequence: [C]TCTTTCTATCCGCCCTCACTT).

### Quantitative analysis of salivirus

The mean viral load of the case group was 6.33×10^4^ copies (1.14×10^0^ − 6.91×10^5^ copies) *vs*. 5.01×10^4^ copies (1.46×10^0^ − 1.86×10^5^ copies) in the control group: a log-normal *t*-test revealed no difference between the cases and controls.

### Association between viral agents and gastroenteritis

The Cox regression analysis revealed a strong association between viral infection and gastroenteritis for RV, NoV/SaV, AdV and AstV, but no association between salivirus infection and gastroenteritis (Table 2). Frequent coinfection of salivirus and NoV/SaV was observed. Clinical symptoms (i.e., rates of fever, frequency of diarrhea and duration of diarrhea) of children infected with NoV/SaV alone *vs*. those coinfected with NoV/SaV and salivirus did not differ (Table 3).

**Table 2:** Multiple factor Cox regression analysis of the association of five viruses with gastroenteritis (*p* < 0.05).

	Significance	OR^a^	95% C.I.^b^ for Exp(B) Lower to Upper
RV	0	167.011	37.795 738.005
HuCV	0	4.778	2.936 7.774
Salivirus	0.774	0.862	0.311 2.387
AstV	0.014	6.415	1.454 28.314
AdV	0	6.159	2.71 13.999

^a^ OR: odds ratio.

^b^ CI: 95% confidence interval.

**Table 3:** Multiple-factor logistic regression analysis of the clinical symptoms of children with gastroenteritis.

	Significance	OR^a^	95.0% C.I. ^b^ for EXP(B) Lower to Upper
Frequency of Diarrhea	0.433	1.134	0.828 1.552
Fever	0.871	0.88	0.188 4.117
Duration of diarrhea	0.864	0.993	0.914 1.078

^a^ OR: odds ratio.

^b^ CI: 95% confidence interval.

## Discussion

Salivirus is a newly identified virus detected in feces in 2009, and has been reported worldwide subsequently [6–10, 17]. Our results confirmed that enteric viruses play an important role in pediatric diarrhea, and further that the most common viral agent associated with gastroenteritis is RV, followed by NoV/SaV, AdV and AstV. The prevalence rates of RV, AdV and AstV were similar to those reported previously [18, 19–21]. However, the detection rate (31.5%) of NoV/SaV in this study was higher than reported previously using RT-PCR [18] or real-time PCR [22, 23], and was in accordance with national viral diarrhea surveillance data from Hunan and Hebei between 2011 and 2012 of China (data not shown). This suggests a high prevalence of calicivirus in these areas of China. Moreover, RV detection peaked during the winter months of both years, in accordance with the results of previous studies. However, the distribution of NoV/SaV, AstV and AdV did not vary according to season in our study.

Since its identification, salivirus has been detected frequently in the stool samples of children with gastroenteritis worldwide, with higher prevalence rates in Nepal (8.6%), Shanghai/China (4.2%), Korea (4%) and Tunisia (3.1%). No positive samples were detected in healthy matched controls [9, 10, 17]. In our study, the detection rate in children with gastroenteritis was 3.5%, similar to other studies; however, the detection rate in healthy children (2.8%) was much higher than in previous studies, possibly due to the small sample sizes employed in the Shanghai/China- (96 samples), Korea- (294 samples) and Tunisia- (96 samples) based studies. Our salivirus-positive case subjects were younger compared with the control group, which might have been due to the moderate sample size and low salivirus detection rate. Furthermore, the detection rates of saliviruses in the two areas (i.e., Lulong and Liuyang County) did not differ, and all salivirus-positive samples were collected in summer months (i.e., between June and September). Lulong County is located in the North of China and Liuyang County in the south of China. This indicates that the virus is strongly associated with seasonality but weakly associated with geographical factors, which is in agreement with previous results pertaining to other enteric viruses, such as enterovirus 71 [24]. Taken together, the evidence indicates that saliviruses circulate widely in China, but with low prevalence and seasonality typical of enteroviruses.

The salivirus detected in stool samples gathered from different areas were very similar (> 90% shared nt identities). However, SaliV-SewBKK, found in sewage in 2012, shared only 83% nt identity with Salivirus A (GenBank GQ184145), but the complete genome of SaliV-SewBKK was not acquired; the sequence lacks a portion of VP0 sequence and the untranslated region (UTR) at the 5′ end. The present study reports a complete genome of a salivirus (SaliV-FHB), detected in stool sample from a child, which shared 99% nt homology with SaliV-SewBKK. The 5′ UTR of SaliV-FHB has a redundant 21/22-bp sequence; it is not yet clear whether this redundant sequence is functional, and if so what that function would be. Therefore, this strain may have a different clinical presentation than other common saliviruses. Genetic diversity among saliviruses may indicate the presence of multiple species, and genotypes could possess different biological properties and antibody neutralization profiles. It is not clear whether SaliV-FHB is genetically indistinguishable from SaliV-SewBKK. Salivirus transmission is likely fecal-oral between persons and/or occurs through contaminated water,

Previous studies have revealed that Aichi viruses and human parechovirus 1 (both of the *Picornaviridae* family) are associated with gastroenteritis [25–28]. Recent seroepidemiological study showed proof of infection with salivirus in children and molecular epidemiological study suggest that childhood infection with salivirus is associated with gastroenteritis [6, 10–11]. However, in the present study there is no difference in detection rate of saliviruses in cases and control subjects. And there is also no significant difference in detection rates of saliviruses by area. Coinfection with other diarrhea-related viruses was very common (62.5%) in the salivirus-positive samples, especially with calicivirus. Furthermore, there is not statisticly difference between the cases and controls in the mean viral load of saliviruses. These evidences suggest salivirus may play no role in gastroenteritis. However, it should be noted that the viral loads were relatively low (i.e., 1.14×10^0^ − 6.91×10^5^ copies) in both the case and control groups. A previous study suggested that norovirus viral loads lower than a threshold level should be regarded as negative; i.e., although norovirus was detected, it was not a causal factor in the disease [29]. This might also be the case with salivirus infection. Therefore, we cannot rule out the possibility that salivirus is related to gastroenteritis when replicating at high viral loads.

However, multivariate conditional Cox regression analysis in the present study suggested salivirus is not a causative agent in child gastroenteritis. The etiological function of other common diarrhea-related viruses (RV, NoV/SaV, AstV and AdV) is clear. Further statistical analysis suggested that salivirus coinfection did not exacerbate the clinical symptoms of gastroenteritis. Further molecular epidemiological studies will be required to determine whether saliviruses are associated with other human diseases or if some specific genotypes of saliviruses (based on capsid protein sequences) are associated with gastroenteritis.

## Supporting Information

S1 Table Primers used for complete genome amplification in the study. “F” stands for forward primer, “R” stands for reverse primer.
(DOC)
