# Supplementary material for: Salivirus in Children and Its Association with Childhood Acute Gastroenteritis: A Paired Case-Control Study
Source: PLoS One. 2015 Jul 20;10(7):e0130977. doi: 10.1371/journal.pone.0130977 (PMC4507861; doi:10.1371/journal.pone.0130977)
Supplement: S1 Table — “F” stands for forward primer, “R” stands for reverse primer. (DOC) [file pone.0130977.s001.doc]

| Primer Name | Sequences (5′-3′) | Position |
| --- | --- | --- |
| Universal Primer A  (5′/3′ RACE kit, Clontech) | CTAATACGACTCACTATAGGGCAAGCAGTGGTATCAACGCAGAGT |  |
| Sali 5race | TGGGAAGTGAGGGCGGATAGAAAGAGAGAG | 211-240 bp |
| Sali walking 2 | TGAGGCCTTGGCGTTGGACAAGGT | 1029-1052 bp |
| Sali walking 1 | GTCAACAACAGGCAGGAGAGCCTG | 1496-1519 bp |
| Sali1 F | TAGCCTACACTCCTCCTTCC | 2517-2536 bp |
| Sali1 R | AGATGTAGAAGGAGGCAGAC | 3162-3182 bp |
| Sali2 F | TCACAGTACCGTTGGTTTGG | 2978-2997 bp |
| Sali2 R | CACAGCATGAGAGAAACACC | 4319-4338 bp |
| Sali3 F | CTGACATCGCTACATCTTCC | 4095-4114 bp |
| Sali3 R | AGGGGAAGGTTAGGAGAGTC | 5524-5543 bp |
| Sali4 F | CACTCCTGTCTCTTGCTTTG | 5400-5419 bp |
| Sali4 R | GGATTGGTAGATGTAGGAGC | 6844-6863 bp |
| Sali5 F | ACTCTCTCCCTGTCCTTTCC | 6658-6677 bp |
| Sali5 R | AAGGAGGGAGCAGCAACTAC | 7916-7935 bp |
| Sali 3race | GTCTCCTCCTGCATGACTGGACCCTAC | 7785-7811 bp |

**Primers used for complete genome amplification in the study**

**Note.** “F” stands for forward primer; “R” stands for reverse primer.
